# A positive COVID-19 test is associated with high mortality in RR-TB-HIV patients

**DOI:** 10.5588/ijtld.21.0010

**Published:** 2021-05-01

**Authors:** E. Mohr-Holland, J. Daniels, B. Douglas-Jones, N. Mema, V. Scott, L. Trivino-Duran, C. Pfaff, J. Furin, P. Isaakidis

**Affiliations:** 1 Médecins Sans Frontières (MSF), Khayelitsha, Cape Town, South Africa; 2 MSF Southern Africa Medical Unit, Observatory, Cape Town, South Africa; 3 City of Cape Town Health Department, Khayelitsha, Cape Town, South Africa; 4 Harvard Medical School, Boston, MA, USA

Dear Editor,

At the time of writing, COVID-19 has killed more than 2.5 million people globally.^1^ A limited number of studies have indicated that TB may be a risk factor for poor outcomes among people diagnosed with COVID-19.^2^,^3^ However, the potential deadly interactions between these two diseases may be overlooked, especially among those with less common forms of TB, such as rifampicin-resistant TB (RRTB). Approximately 500,000 people become sick with RR-TB annually;^4^ therefore this population is likely under-represented in the published cohorts on COVID-19. Khayelitsha, the largest peri-urban informal settlement of Cape Town, South Africa, has been at the epicenter of the HIV, TB/RR-TB and the COVID-19 pandemic.^5^–^7^ We therefore conducted a retrospective study to describe SARS-CoV-2 diagnosis and outcomes among people in Khayelitsha receiving treatment for RR-TB from 1 April to 30 November 2020, using routine programmatic data.

Polymerase chain reaction SARS-CoV-2 results were accessed using the national health laboratory service data system. No data were recorded on the testing indication for SARS-CoV-2 (which changed over the study period from a positive contact or travel history, to being symptom-based, and finally to being risk-based). It is important to note that people with RR-TB were not routinely tested for SARS-CoV-2, and people undergoing SARS-CoV-2 testing were not routinely assessed for TB or RR-TB.

In total, 261 active RR-TB patients were identified, of whom 175 (67%) were HIV-positive. Overall, 75 (29%) patients were newly diagnosed with RR-TB from April 2020 onwards (referred to as recently diagnosed), and the remaining 186 (71%) were diagnosed prior to April 2020. Seventy-five (29%) RR-TB patients received a SARS-CoV-2 test. Twelve (16%) of these were tested for SARS-CoV-2 prior to being investigated for TB; the median time from COVID-19 diagnosis to RR-TB diagnosis among these patients was 7.0 days (interquartile range [IQR] 4.5–8.0). The remaining 63 (84%) patients were investigated for COVID-19 a median of 1.7 months (IQR 0.4–6.4) after their RR-TB diagnosis. Of the 75 recently diagnosed RR-TB patients, 48 (64%) received a SARS-CoV-2 test and six (8%) received a SARS-CoV-2 test the same day.

Of the 75 RR-TB patients tested for SARS-CoV-2, 18 (24%) tested positive and nine (50%) were recently diagnosed. Those who tested positive for SARS-CoV-2 were significantly more likely to be older (median age 43 years vs. 35 years), be diagnosed with and treated for RR-TB in hospital (33% vs. 13% and 17% vs. 2%, respectively) and have diabetes (28% vs. 9%) compared to those who were not tested for SARS-CoV-2 or were SARS-CoV-2-negative (*P* < 0.05, data not shown). Of the 18 RR-TB patients who tested SARS-CoV-2-positive, 11 (61%) died. There were no significant differences in the clinical and demographic characteristics between those who tested SARS-CoV-2-positive and survived versus those who died over the study period (*P* > 0.05), likely owing to the small number of patients who tested SARS-CoV-2-positive (Table). Of the remaining 243 patients who were not tested for SARS-CoV-2 or were SARS-CoV-2-negative, 16 (6.6%) died, indicating that mortality was significantly higher among those who tested SARS-CoV-2-positive (*P* < 0.0001). There were no significant differences in the baseline characteristics of those who died who were SARS-CoV-2-positive versus those who died who were not tested for SARS-CoV-2 or were SARS-CoV-2-negative (Table). Of the 11 SARS-CoV-2-positive patients who died, two were tested for SARS-CoV-2 seven and eight days prior to their RR-TB diagnosis. Of the remaining nine patients, the median time from RR-TB to COVID-19 diagnosis was 2.8 months (IQR 1.8–6.4). Five (56%) of these nine patients were recently diagnosed. The median time from COVID-19 diagnosis to death was 17 days (IQR 0–59). Among the 10 patients with TB who died, four (40%) were culture-negative at the time of death. Overall mortality was significantly higher in the 1 April–30 November time period in 2020 than during the same period in 2019 (27/261, 10.3% vs. 17/303, 5.6%, respectively; *P* = 0.037). The baseline characteristics of those who died did not differ based on year of death (2019 vs. 2020, see Table).

**Table:** Comparison of the clinical and demographic characteristics of the RR-TB patients who died between April and November (a) 2019 and (b) 2020 and of those who died and (c) were not tested for or tested negative for SARS-CoV-2 and (d) tested SARS-CoV-2-positive between April and November 2020; and of those who (e) survived between April and November 2020 and tested SARS-CoV-2-positive

	a) Active in care, April–November 2019 (*n* = 303) *n* (%)	b) Active in care, April–November 2020 (*n* = 261) *n* (%)	c) Active in care and not SARS-CoV-2-positive,* April–November 2020 (*n* = 243) *n* (%)	d) Active in care and tested SARS-CoV-2-positive, April–November 2020 (*n* = 18) *n* (%)	

Died between April and November in the respective year	17 (5.6) a) Died, April–November 2019 (*n* = 17) *n* (%)	27 (10.3) b) Died, April–November 2020 (*n* = 27) *n* (%)	16 (6.6)^†^ c) Died, April–November 2020, not SARS-CoV-2-positive* (*n* = 16) *n* (%)	11 (61)^†^ d) Died, April–November 2020, tested SARS-CoV-2-positive (*n* = 11) *n* (%)	e) Survived, April–November 2020, tested SARS-CoV-2-positive (*n* = 7) *n* (%)
Male	10 (59)	16 (59)	10 (63)	6 (55)	4 (57)
Age at RR-TB diagnosis, years, median [IQR]	42 [32–49]	44 [36–59]	45 [36–59]	44 [36–67]	35 [32–50]
Time of RR-TB diagnosis					
Before 1 April 2020	17 (100)	15 (56)	10 (63)	5 (45)	4 (57)
On or after 1 April 2020	0 (0)	12 (44)	6 (38)	6 (55)	3 (43)
Location of RR-TB diagnosis					
Primary health care facility	13 (76)	17 (63)	10 (62)	7 (64)	5 (71)
Hospital	4 (24)	10 (37)	6 (38)	4 (36)	2 (29)
Location of RR-TB treatment initiation					
Primary health care facility	17 (100)	23 (85)	14 (87)	9 (82)	6 (86)
Hospital	1 (0)	4 (15)	2 (13)	2 (18)	1 (14)
HIV-positive	13 (76)	21 (78)	13 (81)	8 (73)	4 (57)
Baseline CD4, cells/mm^3^ (% HIV-positive)					
<200	10 (77)	13 (62)	9 (69)	4 (50)	3 (75)
200–499	0 (0)	3 (14)	3 (23)	0 (0)	1 (25)
≥500	2 (15)	5 (24)	1 (8)	4 (50)	0 (0)
Unknown	1 (8)	0 (0)	0 (0)	0 (0)	0 (0)
Hypertension	2 (12)	8 (30)	4 (25)	4 (36)	0 (0)
Diabetes	1 (6)	5 (19)	2 (13)	3 (27)	2 (29)
Number of comorbidities (HIV, hypertension and diabetes)					
0	3 (18)	0 (0)	0 (0)	0 (0)	1 (14)
1	12 (71)	20 (74)	13 (81)	7 (64)	6 (86)
2	2 (12)	7 (26)	3 (19)	4 (36)	0 (0)
Previous TB treatment history					
No previous TB treatment history	5 (29)	12 (44)	8 (50)	4 (36)	4 (57)
Previously treated with first-line TB drugs	11 (65)	13 (48)	6 (38)	7 (64)	3 (43)
Previously treated with second-line TB drugs	1 (6)	2 (7)	2 (12)	0 (0)	0 (0)
Site of TB					
Pulmonary TB	17 (100)	25 (93)	15 (94)	10 (91)	7 (100)
Extrapulmonary TB	0 (0)	2 (7)	1 (6)	1 (9)	0 (0)
RR-TB resistance classification					
RR-TB	6 (35)	11 (41)	5 (31)	6 (55)	3 (42)
MDR-TB plus MDR-TB and injectable resistance	7 (41)	11 (41)	9 (56)	2 (18)	2 (29)
Fluoroquinolone-resistant TB	4 (24)	5 (18)	2 (13)	3 (27)	2 (29)

* Not tested for SARS-CoV-2 or tested negative for SARS-CoV-2.

^†^ *P* value for comparison <0.0001.

RR-TB = rifampicin-resistant TB; IQR = interquartile range; MDR-TB = multidrug-resistant TB.

There are several possible explanations for our findings. First, the high mortality may simply be an artifact of limited access to testing for SARS-CoV-2 in the Khayelitsha population, with mild or even moderate infections going undiagnosed. However, the mortality rate of people diagnosed with COVID-19 in South Africa overall (2.7%), suggests that this is not the case.^1^ Second, there could be pathological biological interactions between COVID-19 and RRTB: for example, people with RR-TB may have underlying lung damage and limited pulmonary reserves, thus leading to excess mortality when there is co-infection with SARS-CoV-2. Data from South Africa have shown that COVID-19 severity and mortality rates are higher among people with active TB and RR-TB.^3^,^5^ Third, although there was no significant difference in the rate of HIV co-infection between those who died and tested SARS-CoV-2-positive versus those who died and did not test SARS-CoV-2-positive, it is possible that the added contribution of HIV co-infection to mortality might have heightened the risk of mortality for those with a positive SARS-CoV-2 test.^8^ Fourth, it is possible that the patients in this cohort diagnosed with both diseases received a late diagnosis of COVID-19 and thus were at an advanced stage of the disease, a finding supported by the short time from COVID-19 diagnosis to death. It could be that patients themselves and/or providers attributed respiratory symptoms to TB, and thus were less likely to seek or be offered COVID-19 testing until they were critically ill. Fifth, it is also possible that people with RR-TB were not offered effective interventions for the treatment of moderate to severe COVID-19, either because of concerns about such treatment (i.e., misconceptions about corticosteroids worsening RR-TB) or because of limited health care resources. Sixth, these findings indicate a decrease in access to medical care over this period;^9^ clinic utilisation had decreased and patients were discouraged from visiting primary care facilities and there was less public transport available. Finally, it is possible that these individuals would have died from RR-TB anyway, and COVID-19 was not a factor. However, the rate of mortality in 2020, regardless of SARs-CoV-2 diagnosis was significantly higher than that over the same period in 2019, suggesting a potential pathologic contribution of COVID-19.

This study was limited as a result of the small sample size and the retrospective, single-setting design. As a result of the limited programmatic data, we were not able to evaluate differences in the clinical and demographic characteristics between those who tested SARS-CoV-2-positive who survived or died over the study period, to identify factors associated with mortality. Furthermore, there were no autopsies conducted to determine the cause of death. Finally, there was a lack of data on the indication for SARS-CoV-2 testing, which changed over the study period.

Our results highlight the need for a focused investigation into the impact of COVID-19 on people with RR-TB. Also, given the likelihood of increased excess mortality in people with both infections, we recommend that vaccination against SARs-CoV-2 be prioritised in this population. First, there is a need for routine and systematic screening of all people with RR-TB for signs and symptoms of COVID-19 during their interactions with the health system. A concern highlighted in this study is that of the 75 recently diagnosed RR-TB patients, only 48 (64%) received a SARS-CoV-2 test despite the presence of respiratory symptoms. Second, because of the overlap in symptoms for the two diseases, both patients and providers could mistakenly attribute COVID-19 symptoms to TB. We therefore suggest there should be a low threshold for SARS-CoV-2 testing of people living with RR-TB. Third, while maintaining measures to decrease in-person clinical visits by providing longer medication refills and telephonic support, extra attention should be given to ensure access to hospitalisation is not decreased for those who need it. There may be the need for early hospitalisation and compassionate, integrated, and comprehensive management of people with RR-TB who are diagnosed with COVID-19. It would be tragic if recent gains in reducing the morbidity and mortality of RR-TB were undone by delays, or a lack of access to effective therapy for those found to have COVID-19.^10^ Modelling studies have indicated that COVID-19 is linked to in excess of 400,000 deaths from TB in 2020 alone.^11^ While much of this is ascribed to the strain COVID-19 has put on health systems leading to a decrease in TB diagnosis and treatment,^9^,^12^ our findings hint at other potential impacts of these two diseases.
